# Associations between parental impulsivity and child body mass index

**DOI:** 10.1186/s40064-016-3048-x

**Published:** 2016-08-26

**Authors:** Ester F. C. Sleddens, Gill A. ten Hoor, Gerjo Kok, Stef P. J. Kremers

**Affiliations:** 1 Department of Health Promotion, NUTRIM School for Nutrition and Translational Research in Metabolism, Maastricht University Medical Center+, P.O. Box 616, 6200 MD Maastricht, The Netherlands; 2 Department of Human Biology, NUTRIM School for Nutrition and Translational Research in Metabolism, Maastricht University Medical Center+, Maastricht, The Netherlands; 3 Department of Work and Social Psychology, Maastricht University, P.O. Box 616, 6200 MD Maastricht, The Netherlands

**Keywords:** Adolescents, Body mass index, Child, Impulsivity, Parents

## Abstract

**Objective:**

The aim of this study was to examine the association between parental impulsivity and (12–15 year old) child body mass index (BMI).

**Methods:**

In total, 300 parents completed a survey regarding their own impulsivity level (Barratt impulsiveness scale) and that of their child (impulsivity scale of the temperament in middle childhood questionnaire), and supplied details of their own and their child’s height and weight. Partial correlations were computed to assess relationships between both parental and child impulsiveness scores and child BMI z-scores, independent of parental BMI. Mediation analyses were performed to assess the potential mediating role of child impulsivity on the relationship between parental impulsivity and child BMI z-score.

**Results:**

For daughters, parental impulsivity was significantly correlated with BMI z-score. Parent-reported child impulsivity was not related to child BMI z-score, and no evidence was found for a mediating effect of parent-reported child impulsivity on the relationship between parental impulsivity and child BMI z-score.

**Conclusion:**

There is a stronger association between parental impulsivity and child BMI z-score than between child impulsivity and child BMI z-score. The relationship between parental impulsivity and-child BMI z-score could possibly be explained by parenting styles and practices. The potentially mediating role of parenting should be taken into account in future studies investigating the role of personality in children becoming overweight or obese.

**Electronic supplementary material:**

The online version of this article (doi:10.1186/s40064-016-3048-x) contains supplementary material, which is available to authorized users.

## Significance

### What is already known

Although parental impulsivity may play an important role in the positive association between child impulsivity and BMI z-score there is a lack of studies exploring the role of parental impulsivity on childhood overweight.

### What this study adds

The current study did not confirm findings from previous studies linking child impulsivity to child BMI z-score. This study demonstrates that parental impulsivity scores are associated with daughters’ BMI z-scores. This relationship could possibly be explained by parenting styles and practices. No evidence for a mediating role of parent-reported child impulsivity was found.

## Background

Parental impulsivity may play an important role in the positive association between child impulsivity and BMIz-score. Numerous studies have been conducted assessing the influence of a child’s temperament (i.e., individual differences in behavioral patterns) on the development of weight status (e.g., Braet et al. 2007; Anzman-Frasca et al. 2012; Thamotharan et al. 2013; Bergmeier et al. 2014). With regard to child impulsivity, a temperamental trait, the evidence thus far suggests that high impulsivity in childhood is associated with being overweight and obese (Thamotharan et al. 2013). There are also indications that children with attention-deficit/hyperactivity disorder (ADHD), in which impulsivity is common, are heavier than children not diagnosed with ADHD (Cortese et al. 2008; Flier et al. 2013). It has been suggested that impulsive behaviors could contribute to excessive food intake (Davis 2010) and unhealthy snack consumption (Scholten et al. 2014). A Dutch study investigating parents of 6–13 year olds supported this notion by demonstrating that child impulsivity was indirectly associated with child body mass index (BMI) through overeating (Van den Berg et al. 2011). Moreover, a recent review study found that food-related impulsivity was more prevalent in obese children (Schag et al. 2013).

**Fig. 1:**
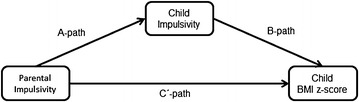
Model of the relationship between parental impulsivity and child BMI: potential mediating role of child impulsivity

Although several studies have been carried out to investigate the influence of child impulsivity on weight status, there is a lack of studies exploring the role of parental impulsivity on childhood overweight. Impulsive traits may be inherited, but are likely to be influenced over time by maturation and experience, including parenting processes. Therefore, parental impulsivity may also influence a child’s BMI z-score via the parenting processes. It is well-known that parents play a key role in influencing the development of their child’s weight status (e.g., Monasta et al. 2010; Cislak et al. 2012). It is also recognized that mothers and fathers have a differential role in influencing child weight-related outcomes, and, although the exact mechanisms remain unclear, these effects are likely to differ with regard to boys and girls (Wake et al. 2007; Berge et al. 2010; Jansen et al. 2013; Gevers et al. 2015). In this study, we assess relationships between parental impulsivity and children’s BMI z-score, and examine the potential mediating role of parent-reported child impulsivity on this relationship (see Fig. 1 for a visual representation of our conceptual model).

## Methods

Following recent requests for full disclosure (Peters et al. 2012), all research materials, data, analyses and output are available in a combined.rar archive labelled as Additional file 1. This study (as well as the consent procedure) was approved by the Ethical Review Committee Psychology and Neuroscience (ERCPN), Maastricht University, the Netherlands.

### Participants

In total, 600 parents of 12–15 year old adolescents were randomly invited to participate in our study via Flycatcher. The parents that were invited included both mothers and fathers. Flycatcher, a representative online panel for the Dutch population (http://www.flycatcher.eu/; ISO 26362 and ISO20252; Dutch quality label, certifying that the panel can be used for social-scientific research) has 1300 registered parents of adolescents in the age range 12–15 years. After dropout (wrong e-mail address (*N* = 10) and the exclusion of questionable data (the use of straight-lining, or other patterned response strategies; nonsense answers on open questions, *N* = 23), 314 parents completed the study (53.2 % response rate).

### Procedure and measures

Before participation in this computer study, all parents provided informed consent by clicking on the appropriate button. They were instructed that all questions related to their youngest child in the age range 12–15 years. No children participated in this study. Information about gender and age of both the parent and child, and the highest level of education attained by the parent (categorized into low—no education or primary education; medium—intermediate/high general secondary education or intermediate vocational education; and high—college degree or higher) had already been collected by Flycatcher. In addition to these background characteristics, parents were asked questions about both their own and their child’s impulsiveness and anthropometrics (i.e., height and weight).

### Impulsivity in children

To measure impulsivity in children, we used the impulsivity scale from the parent-reported version of the temperament in middle childhood questionnaire (TMCQ) (Simonds and Rothbart 2004), adapted from the children’s behavior questionnaire (CBQ) (Rothbart et al. 2001). The CBQ is an instrument used to assess three broad dimensions of temperamental traits including surgency/extraversion, negative affectivity, and effortful control in children between 3 and 6 years old (Rothbart et al. 2001). The TMCQ has previously been validated against the CBQ in a Dutch sample (Sleddens et al. 2013). This validation study supported the applicability of the TMCQ as compared with all three previously mentioned temperamental traits of the CBQ. Impulsivity, as measured by the TMCQ, is considered to be a subfactor of surgency/extraversion. Parents were asked to respond to 13 statements describing their child’s impulsive behaviors (e.g., ‘My child decides what she/he wants very quickly and then goes after it’). They were asked to choose the answer that applied to their child the most, using a 5-point Likert scale ranging from 1 (*Never*) to 5 (*Always*). Cronbach’s alpha for the TMCQ scale in the present sample was 0.89. Corrected-item total correlations (CITCs) ranged from 0.41–0.73 for 13 items, with an average of 0.58, indicating homogeneity of the items (Nunnally and Bernstein 1994).

### Impulsivity in parents

For the measurement of impulsivity in parents, we used the 30-item Barratt impulsiveness scale (BIS-11) (Patton et al. 1995). Sample items include ‘I do things without thinking’, and ‘I plan tasks carefully (reverse coded)’. Cronbach’s alpha for the BIS in the present sample was 0.82. However, three items had CITCs below the critical cut-off point of 0.15, defined by Nunnally and Bernstein (1994); ‘I make-up my mind quickly’ (CITC = 0.11), ‘I am happy-go-lucky’ (CITC = −0.03), and ‘I change residences’ (CITC = 0.13). The Cronbach’s alpha of the resulting 27-item scale was 0.83, with CITCs ranging from 0.15 to 0.56, with an average of 0.37.

### Body mass index

Parents were asked to indicate (specified to one decimal place) their own and their child’s weight (kg) and height (cm), which we used to calculate BMI (in kg/m^2^). Each child’s BMI was then recoded into an age and gender-specific BMI z-score and compared to the national reference population (Fredriks et al. 2000). A BMI z-score >85th percentile was considered to indicate overweight and a BMI z-score >95th percentile was considered to indicate obesity (Barlow 2007). BMI z-scores <−5.0 or >5.0 were considered to be unrealistic, as advised by the WHO (De Onis et al. 2007). Only two children had a BMI z-score below −5 and were therefore removed from further analyses involving child BMI z-scores. Additionally, 12 children had missing values for exact day of birth. We also removed data from these children from all further analyses.

### Data analyses

IBM SPSS statistics 20 was used to analyze the data. Descriptive analyses—frequencies (*N*), means and standard deviations (*SD*)—were calculated to assess the background characteristics of the sample (i.e., parent and child age and BMI (z-score), and parental educational level). Associations between both parental and child impulsiveness scores and child BMI z-scores were examined by computing partial correlations for the total sample (*N* = 300), for mothers and fathers separately, and for sons and daughters separately. The partial correlations were controlled for the potentially confounding variable of parental BMI. Additionally, single mediation analyses were carried out using the PROCESS software including the bootstrapping method with bias-corrected confidence estimates (Mackinnon et al. 2004; Preacher and Hayes 2004) in order to test the direct and indirect associations linking parental impulsivity scores to child BMI z-scores. Bootstrapping, a non-parametric sampling procedure, was used to assess the significance of indirect effects. In the present study, the 95 % confidence interval of the indirect effects was obtained with 5000 bootstrap resamples; results are statistically significant when 95 % confidence intervals do not overlap zero. This procedure was repeated for different groups (i.e., total sample, fathers and mothers, and sons and daughters). Also the mediation analyses were controlled for the potentially confounding variable of parental BMI.

## Results

Key characteristics of the sample (*N* = 300) are depicted in Table 1. An approximately equal number of males and females participated in the study; 44.3 % (*N* = 133) of the questionnaires were completed by mothers and 55.7 % (*N* = 167) were completed by fathers. Mean (*SD*) age of the participants was 45.8 (4.8) years. The majority of the participants reported medium or higher levels of education: 44.7 % reported having intermediate/high general secondary education or intermediate vocational education, and 32.3 % reported having a college degree or higher. Gender of the children was also equally divided (49.7 % girls). The mean age of the children was 13.4 (*SD* = 1.1) years. With regard to weight status, 62.0 % of the parents were overweight or obese, and for children this percentage was 21.7 %. These percentages seem representative for the Dutch population except for a slight overrepresentation of highly educated parents.

**Table 1:** Background characteristics of the sample (N = 300)

	Parent	Child
Gender (female: male)	133:167	149:151
Mean age in years (SD)	45.8 (4.8)	13.4 (1.1)
Education level		
Low (%)	69 (23.0 %)	–
Medium (%)	134 (44.7 %)	–
High (%)	97 (32.3 %)	–
Mean BMI (z) (SD)^a^	26.74 (4.49)	−0.05 (1.32)
Underweight (%)	3 (1.0 %)	36 (12 %)
Normal-weight (%)	111 (37.0 %)	199 (66.3 %)
Overweight (%)	125 (41.7 %)	36 (12 %)
Obese (%)	61 (20.3 %)	29 (9.7 %)

All values are *N*’s, unless otherwise indicated

Education level: low = no, or primary education; medium = intermediate/high general secondary education or intermediate vocational education; high = college degree or higher

^a^For the parents a BMI score was calculated; for the youngsters a BMI z-score was calculated

Table 2 presents partial correlation coefficients (controlled for parental BMI) between both parental and child impulsivity scores and child BMI z-scores for different groups (i.e., total sample and sample split by parental and child gender). With regard to the correlation between parental impulsivity and child impulsivity, we found significant positive relationships for the total sample, (*r* ranging from 0.18 to 0.23, *p* < 0.05). However, these positive relations are caused by the significant correlations between maternal impulsivity and child impulsivity (*r 0*.33–0.47, p < 0.01) while the correlations between paternal impulsivity and child impulsivity were not significant (*r* = 0.12–0.14, *p* > 0.12). A positive significant correlation was only found between parental impulsivity scores and daughters’ BMI z-scores (*p* < 0.05). The correlation between parental impulsivity scores and child BMI z-scores was marginally significant for the total sample. The relationship between maternal impulsivity scores and child BMI z-scores was also marginally significant. Child impulsivity scores were only marginally significantly related to daughters’ BMI z-scores.

**Table 2:** Partial correlations between parental impulsivity, child impulsivity and child BMI z-score

	Child impulsivity			Child BMI z-score		
	T	♂	♀	T	♂	♀
Parent impulsivity						
Total sample	*0.23 (<0.001)*	*0.27 (0.001)*	*0.18 (0.03)*	0.10 (0.10)^a^	0.01 (0.92)	0.*19 (0.02)*
Fathers	0.12 (0.12)	0.14 (0.20)	0.12 (0.28)	0.05 (0.56)	−0.10 (0.37)	0.15 (0.18)
Mothers	*0.40 (<* *0.001)*	*0.47 (<* *0.001)*	*0.33 (0.007)*	0.16 (0.07)^a^	0.08 (0.50)	0.24 (0.06)^a^
Child impulsivity						
Total sample				0.05 (0.41)	−0.04 (0.65)	0.15 (0.08)^a^
Boy				0.05 (0.56)	−0.05 (0.65)	0.17 (0.12)
Girl				0.6 (0.52)	−0.00 (0.97)	0.12 (0.33)

All values are *r* (significance level). In italics *p* < 0.05

^a^Marginally significant findings *p* < 0.10; analyses controlled for parental BMI

Finally, the results of the mediation analyses to assess the potential mediating role of child impulsivity on the relationship between parental impulsivity scores and child BMI z-scores after controlling for parental BMI are presented in Table 3 (see Fig. 1 for a description of the different pathways). Child impulsivity was not found to be a mediator in any of the groups (i.e., total sample, fathers and mothers, or sons and daughters). For four out of the five groups (all except fathers), the A path linking parental impulsivity to child impulsivity was significant, indicating that parental impulsivity was positively associated with child impulsivity. Analyses further revealed that parental impulsivity scores were significantly associated with daughters’ BMI z-scores after controlling for child impulsivity (*ß* = 0.74, *SE* = 0.36, *t* = 2.07, *p* = 0.04), indicating a direct effect of parental impulsivity on daughters’ BMI z-scores. Indirect effects of parental impulsivity on child BMI z-scores were non-significant in all groups. Parental BMI did not act a as covariate, only on the relationship between parental impulsivity and children’s BMI in the total group (see Additional file 1).

**Table 3:** Mediation analyses: a mediating role of child impulsivity on parent impulsivity scores and child body mass index z-scores

Sample	*N*		
Total sample	300	***A path*** B path	***β*** ***=*** ***0.54, SE*** ***=*** ***0.13, t*** ***=*** ***4.06, p*** ***<*** ***0.001*** *β* = 0.05, *SE* = 0.11, *t* = 0.47, *p* = 0.64
		Direct effect (C’ path)	*β* = 0.40, *SE* = 0.27, *t* = 1.50, *p* = 0.13
		Indirect effect (a x b)	*β* = 0.02, boot *SE* = 0.06, *CI SE* = −0.09 to 0.15
Fathers	167	A path	*β* = 0.27, *SE* = 0.17, *t* = 1.55, *p* = 0.12
		B path	*β* = 0.08, *SE* = 0.16, *t* = 0.51, *p* = 0.61
		Direct effect (C’ path)	*β* = 0.19, *SE* = 0.36, *t* = 0.52, *p* = 0.61
		Indirect effect (a × b)	*β* = 0.02, boot *SE* = 0.05, *CI SE* = −0.04 to 0.20
Mothers	133	***A path***	***β*** ***=*** ***0.99, SE*** ***=*** ***0.20, t*** ***=*** ***4.96, p*** ***<*** ***0.001***
		B path	*β* = −0.01, *SE* = 0.17, *t* = −0.09, *p* = 0.93
		Direct effect (C’ path)	*β* = 0.71, *SE* = 0.42, *t* = 1.70, *p* = 0.09*
		Indirect effect (a × b)	*β* = −0.01, boot *SE* = 0.16, *CI SE* = −0.37 to 0.28
Son	151	***A path***	***β*** ***=*** ***0.64, SE*** ***=*** ***0.18, t*** ***=*** ***3.63, p*** ***<*** ***0.001***
		B path	*β* = −0.08, *SE* = 0.17, *t* = −0.49, *p* = 0.62
		Direct effect (C’ path)	*β* = 0.09, *SE* = 0.39, *t* = 0.24, *p* = 0.81
		Indirect effect (a × b)	*β* = −0.05, boot *SE* = 0.10, *CI SE* = −0.30 to 0.12
Daughter	149	***A path***	***β*** ***=*** ***0.44, SE*** ***=*** ***0.19, t*** ***=*** ***2.26, p*** ***=*** ***0.03***
		B path	*β* = 0.21, *SE* = 0.15, *t* = 1.38, *p* = 0.17
		***Direct effect*** (C’ path)	***β*** ***=*** ***0.74, SE*** ***=*** ***0.36, t*** ***=*** ***2.07, p*** ***=*** ***0.04***
		Indirect effect (a × b)	*β* = 0.09, boot *SE* = 0.08, *CI SE* = −0.02 to 0.32

In bolditalics *p* < 0.05; * marginally significant findings *p* < 0.10 and *p* > 0.05

*A path* parental impulsivity on child impulsivity; *B path* child impulsivity on child BMI; direct effect (*C’ path*): parental impulsivity on child BMI after controlling for child impulsivity; indirect effect (a × b): parental impulsivity on child BMI; all analyses controlled for parental BMI

## Discussion

This study examined associations between parental impulsivity and child BMI z-score, and assessed parent-reported child impulsivity as a potential mediator of this association. Analyses revealed that parental impulsivity scores were associated with daughters’ BMI z-scores. Evidence for a mediating role of parent-reported child impulsivity was not found.

The possible mediating role of parenting should be examined in future studies. Although we did not assess aspects of parenting in our study, evidence is available suggesting that parental impulsivity contributes to child weight status through parenting processes. Parents can play a pivotal role in influencing their child’s BMI through their personality characteristics, and the way in which their personality is translated into parenting. Findings from a meta-analytic review on associations between the major personality factors and parenting showed that effect sizes were significant and robust (Prinzie et al. 2009), in line with the notion that personality affects parenting. This finding has been confirmed in more recent studies (e.g., De Haan et al. 2012; Sleddens et al. 2014). For example, De Haan et al. (2012) reported that the temperamental traits of agreeableness and extraversion were important predictors of parental over-reactivity and warmth. Sleddens et al. (2014) showed that parenting constructs such as nurturance, structure and behavioral control were positively correlated with the temperamental traits of extraversion, agreeableness, conscientiousness and openness to experience. Parenting constructs such as coercively controlling and overprotecting home environments were positively related to parental neuroticism. Since multiple studies have shown that an authoritative parenting style is associated with healthier child outcomes, including lower BMI values (Sleddens et al. 2011; Pinquart 2014), it is likely that parental impulsivity contributes to child weight status through parenting processes.

In the present study, differences were found among fathers and mothers, and sons and daughters. For the relationship between parental impulsivity and child BMI z-score, there was only a significant positive relationship for daughters. It could be that impulsive girls with impulsive parents are more prone to disruptive eating behaviors which may in turn lead to increases in BMI z-scores. The current study did not confirm findings from previous studies linking child impulsivity to child BMI z-score (e.g., Bodell et al. 2012; Thamotharan et al. 2013). It could be that the effect of a child’s temperament on the development of his/her BMI z-score depends somewhat on parenting styles and practices, as reported by Zeller et al. (2008); Wu et al. (2011). Children with a difficult temperament and with mothers who scored low on nurturing were found to have a significantly higher risk of becoming overweight or obese (Zeller et al. 2008; Wu et al. 2011). As well as the influence of child temperament, future studies should consider the potential role of additional factors on child BMI z-score. For instance, the link between child impulsivity and BMI could be indirect. Braungart-Rieker et al. (2014) found that child impulsivity was only linked to child BMI indirectly through children’s food approach eating styles. However, secondary analyses on additional data of our study did not show evidence for the mediating mechanism of either parental BMI or child physical activity levels on the link between child impulsivity and child BMI z-scores, also not for the gender-related sub-groups. With regard to future research initiatives, it may also be beneficial to measure the child’s impulsivity directly. To specify, Thamotharan et al. (2013) conducted a meta-analytic review on the relation between child impulsivity and weight status and findings revealed a moderate effect size, such that impulsivity was greater among overweight and obese children, relative to healthy weight children. They conducted a sub-group analysis to see whether the effect sizes differed depending on type of measure used (i.e., behavioral or self-report). A large effect size was found for behavioral measures of impulsivity compared to a small effect size for self-report measures. The findings of the current study should be seen in light of these findings. Usually self-report measures appear to assess broad domains of behaviors over a larger time interval and therefore also more prone to several forms of biases. This is also consistent with results that behavioral and self-report measures of impulsivity are usually not highly correlated (Dougherty et al. 2005).

One advantage of our study was that males and females were equally represented. Several limitations of the present study should, however, also be acknowledged. All of the variables we measured were reported by parents. This could at least in part explain why we did not find a relationship between child impulsivity and child BMI z-score. Our study may have been prone to social desirability effects, which may have diminished any association between impulsivity and child BMI z-score. Highly educated parents were somewhat overrepresented in our sample. Therefore, we must be cautious in generalizing our study findings. Additionally, our cross-sectional design limits the causal conclusions that can be drawn. Future studies are warranted to explore the influence of dynamic parenting processes as well as parent and child temperament on a child’s risk of becoming overweight or obese. As our results demonstrate, such studies should also take gender differences into account.

The association between parental impulsivity and child BMI z-score is stronger than the association between parent-reported child impulsivity and child BMI z-score.

## Additional file

10.1186/s40064-016-3048-x Full disclosure of data, analyses and output.
